# Biowaste-grown live microbial feed additive sustainably and significantly cut enteric methane emissions in Indian livestock

**DOI:** 10.1038/s41598-025-29303-9

**Published:** 2026-01-13

**Authors:** Varunkumar S. Asediya, Makbul A. Shekh, Kalpesh K. Sorathiya, Paresh R. Pandya, Srinivas M. Duggirala, Aashish C. Patel, Urszula Czarnik, Chandra S. Pareek, Sanjay B. Jadhao

**Affiliations:** 1 Animal Nutrition Research Station, College of Veterinary Science and Animal Husbandry, Anand, Kamdhenu University, Gandhinagar, India; 2 Institute of Veterinary Medicine, Faculty of Biological and Veterinary Sciences, Nicolaus Copernicus University, Toruń, 87-100 Poland; 3 Department of Microbiology, Gujarat Vidyapith, Ahmedabad, India; 4 Department of Animal Genetics and Breeding, College of Veterinary Science and A.H., Anand, Kamdhenu University, Gandhinagar, India; 5 Department of Pig Breeding, Faculty of Animal Bioengineering, University of Warmia and Mazury in Olsztyn, ul. M. Oczapowskiego 5, Olsztyn, 10-719 Poland; 6 ICAR–Central Institute of Fisheries Education, Mumbai, India; 7 International Nutrition Inc., 7706 I Plaza, Omaha, NE 68127 USA

**Keywords:** Enteric methane mitigation, Live microbial feed additive, Feed efficiency, IPCC Tier-2 modelling, Circular bioeconomy, Sustainable livestock (India), Antimicrobials, Archaea, Bacteria, Microbial communities, Biological techniques, Microbiology, Climate sciences, Environmental sciences, Health care

## Abstract

Ruminant enteric methane, the largest agricultural source of CH₄, is a key target in global climate policies. We developed a biowaste-derived live fed microbial (LFM) from fruit- and vegetable residues and evaluated its potential as a scalable intervention to reduce enteric methane while improving animal performance. In controlled in vitro assays and a 98 days in vivo feeding trial in bovine calves (*n* = 15), LFM at 2% dietary inclusion (dry-matter basis) improved feed efficiency by 30.9%, reduced modelled methane emissions by 25.2%, increased total volatile fatty acids by 45.5%, and lowered NH₃–N by 28.4%. At 3% inclusion, feed efficiency improved by 25.5%, methane emissions decreased by 30.4%, total VFA increased by 43.0%, and NH₃–N declined by 11.7%. Methane abatement was estimated by integrating in vitro and in vivo measurements using an empirically fitted conversion factor and Tier-2–compatible intake models. The IPCC (2006) Tier-2 equivalents indicated ~19% reduction. Scaling to India’s livestock herd suggested abatement of 15.4 Mt CH₄ yr⁻¹ (432.3 Mt CO₂-eq yr⁻¹; GWP₁₀₀ = 28) under full adoption, corresponding to ~US$494.1 million annually under the carbon-price assumption used. These findings position biowaste-derived LFM as a circular-economy feed technology capable of simultaneously improving productivity and reducing enteric methane emissions at scale.

**Supplementary Information:**

The online version contains supplementary material available at 10.1038/s41598-025-29303-9.

## Introduction

Methane (CH₄) has a 100-year global warming potential approximately 27–29 times that of carbon dioxide (CO₂)^1–3^ and is produced in the rumen during enteric fermentation by methanogenic archaea that reduce CO₂ using hydrogen^4,5^. Enteric CH₄ typically represents ~2–12% of gross energy intake, reducing feed efficiency and contributing to the climate footprint of ruminant systems^6,7^. Nitrous oxide (N₂O) has a GWP₁₀₀ of ~273^8^ and arises from nitrification and denitrification of ammonium in manure and soils; reactive-nitrogen losses drive downstream eutrophication^9^. Together, CH₄ and N₂O account for over 60% of agricultural greenhouse-gas emissions^10^, motivating interventions that integrate hydrogen-sink strategies with improved nitrogen-use efficiency to align climate and productivity goals^11^.

Multiple nutritional and microbial strategies can mitigate enteric CH₄ in ruminants. Optimising the roughage-to-concentrate ratio shifts fermentation towards propionate, a hydrogen sink that curtails methanogenesis^12,13^. Increasing rumen-undegradable (protected) protein can alter rumen function and lower CH₄^14,15^. Feed additives such as methanogenesis inhibitors (for example, 3-nitrooxypropanol)^16^, electron acceptors (nitrate, sulphate), ionophores (monensin), plant bioactives (tannins, saponins), dietary lipids, and exogenous enzymes act by inhibiting methanogens, providing alternative hydrogen sinks, or restructuring the rumen microbiome^17^. Microbial interventions, including defaunation and the use of prebiotics, probiotics, or engineered strains can also reduce CH₄^18^. Among these approaches, live feed microbial (LFM) is cost-effective, antibiotic-free, aligned with sustainability goals, and widely studied as enteric-methane modifiers^19–25^. *Saccharomyces cerevisiae* enhances fibre degradation and promotes propionate formation, with in vivo cattle and sheep trials reporting ~8–16% reductions in methane emissions^19,20^. *Megasphaera elsdenii*, a lactate-utilising bacterium, promotes propionate formation as an alternative hydrogen sink and can reduce methane when supplemented with high-grain diets in feedlot cattle^21,22^. *Clostridium butyricum*, a butyrate-producing anaerobe, shifts ruminal fermentation towards higher butyrate production and altered acetate to propionate ratios^23^. Supplemented strains such as *Lactobacillus acidophilus* and *Bacillus subtilis* can improve ruminal redox balance, enhance ammonia utilisation, and shift the microbiome to disfavour methanogens, thereby lowering CH₄^24,25^.

To harness the methane-mitigating potential of such species, we formulated a multistrain LFM using solid-state fermentation (SSF), which utilises agro-industrial by-products (for example, fruit and vegetable peels and pulp) as substrates to cultivate beneficial microbes^26,27^. In 2022, ~1.05 billion tonnes of food waste were generated globally, contributing an estimated 8–10% of anthropogenic greenhouse-gas emissions (about 5 Gt CO₂-eq)^28^. In India, post-harvest losses in 2020–2022 were valued at about US$18.5 billion, with ~19–40 Mt of fruit and vegetable waste generated annually, representing around 16% of agricultural output^10,29^. These under-utilised streams can be repurposed as agricultural inputs, reintegrating organic waste into productive systems within a circular-economy model and reducing dependence on finite resources^30^.

The initial in vitro phase evaluated the effects of the LFM on digestibility and methane production. The in vivo phase then assessed growth performance, feed conversion, nitrogen dynamics, and haematological parameters in post-weaned calves. In vivo methane output was estimated using a validated predictive equation. We scaled the findings to the national herd using livestock-census data to estimate abatement potential and associated the economic value.

## Results

### In vitro dry matter digestibility and in vitro methane production

**Fig. 1:**
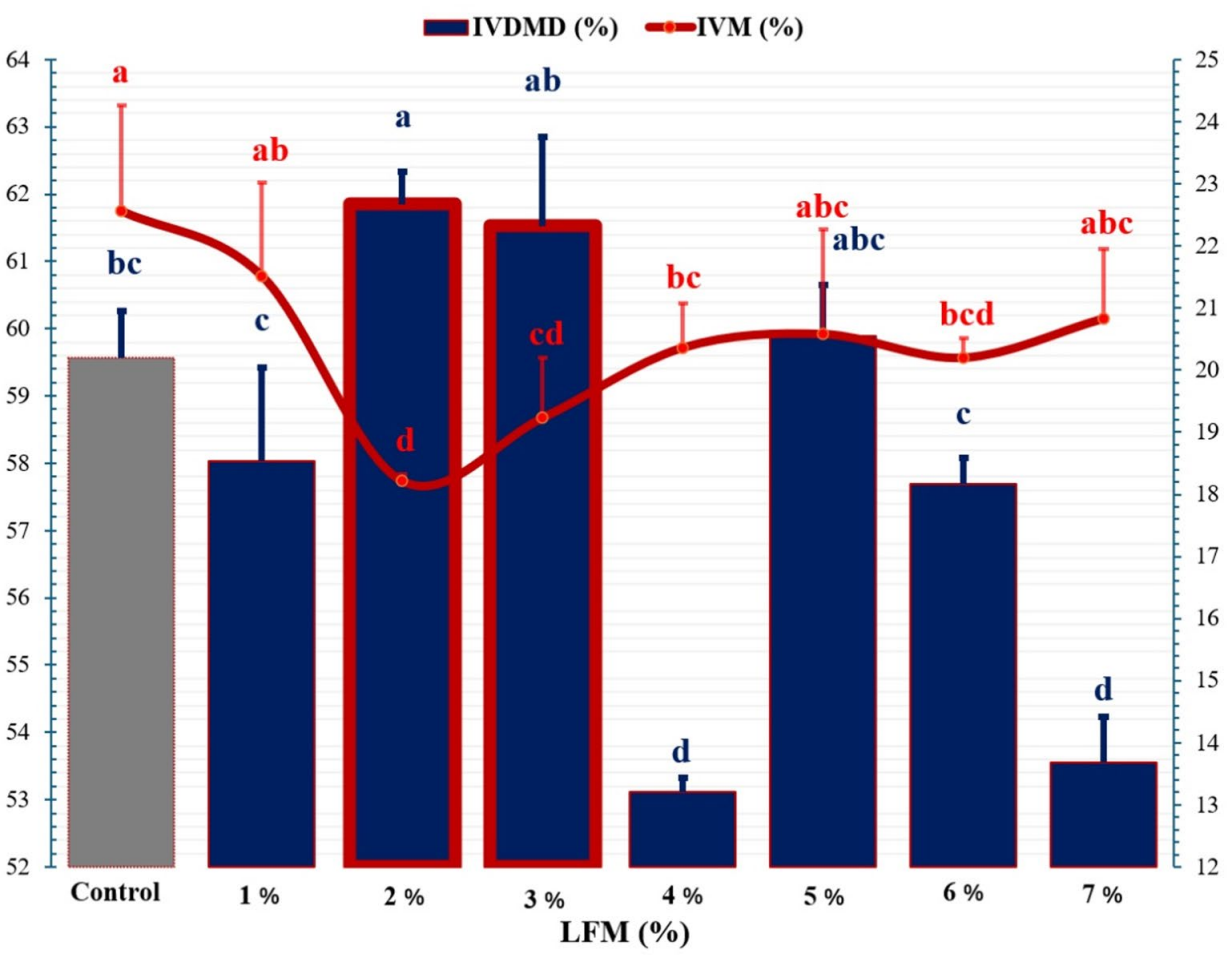
Effects of LFM on in vitro dry-matter digestibility (IVDMD) and in-vitro methane (IVM) production. Batch incubation of TMR with 0–7% LFM (*n* = 3 bottles per dose); IVM expressed as CH₄ per 100 mg of digestible dry matter. Bars show mean ± s.e.m. Letters (a–d) indicate pairwise differences at *P* < 0.05.

Supplementation with LFM altered ruminal fermentation and methane production in a dose-dependent manner (Fig. 1). At 2% and 3% dietary inclusion, in vitro dry matter digestibility (IVDMD) increased by 3.8% and 3.3%, respectively, compared with the control (*P* < 0.05). Methane production decreased by 21.3% at 2% inclusion, while the 4% level yielded the largest reduction per 100 mg of digestible dry matter (DDM), achieving a 48.5% decrease. In vivo, dry-matter intake per unit metabolic body weight (BW⁰·⁷⁵) was reduced by 9.9% and 7.4% at 2% and 3% inclusion (*P* < 0.05). These findings indicate substantial efficiency gains even at modest inclusion levels.

### Dry-matter intake and ruminal fermentation

**Fig. 2:**
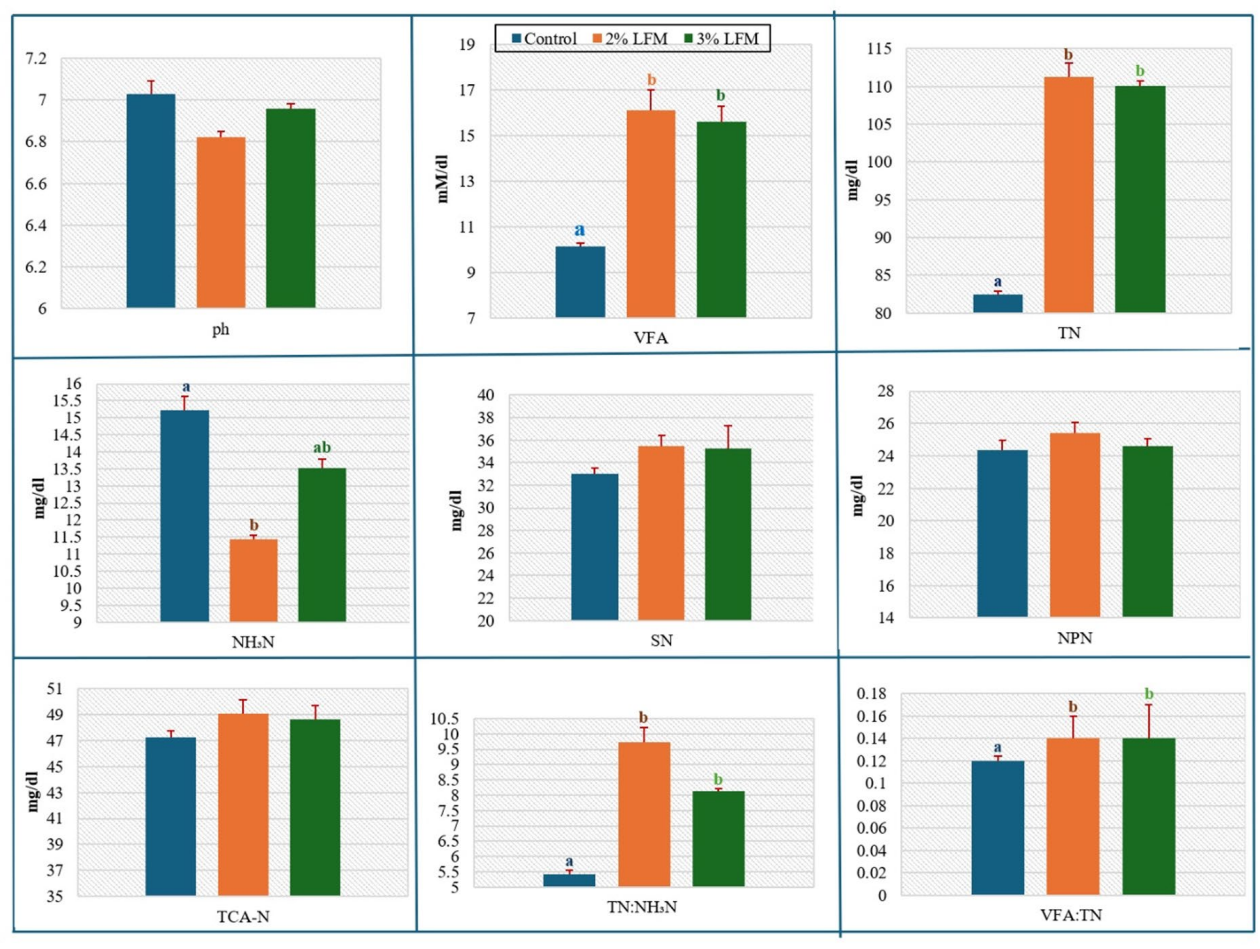
Effects of LFM on rumen fermentation parameters. Variables include pH; total volatile fatty acids (VFA, mmol L⁻¹); total nitrogen (TN, mg L⁻¹); soluble nitrogen (SN, mg L⁻¹); non-protein nitrogen (NPN, mg L⁻¹); trichloroacetic-acid–precipitated nitrogen (TCA-N, mg L⁻¹); ammonia nitrogen (NH₃-N, mg L⁻¹); TN:NH₃-N; and VFA ratio. Bars show mean ± s.e.m. Letters (a, b) indicate *P* < 0.05. *n* = 5 calves per group.

**Fig. 3:**
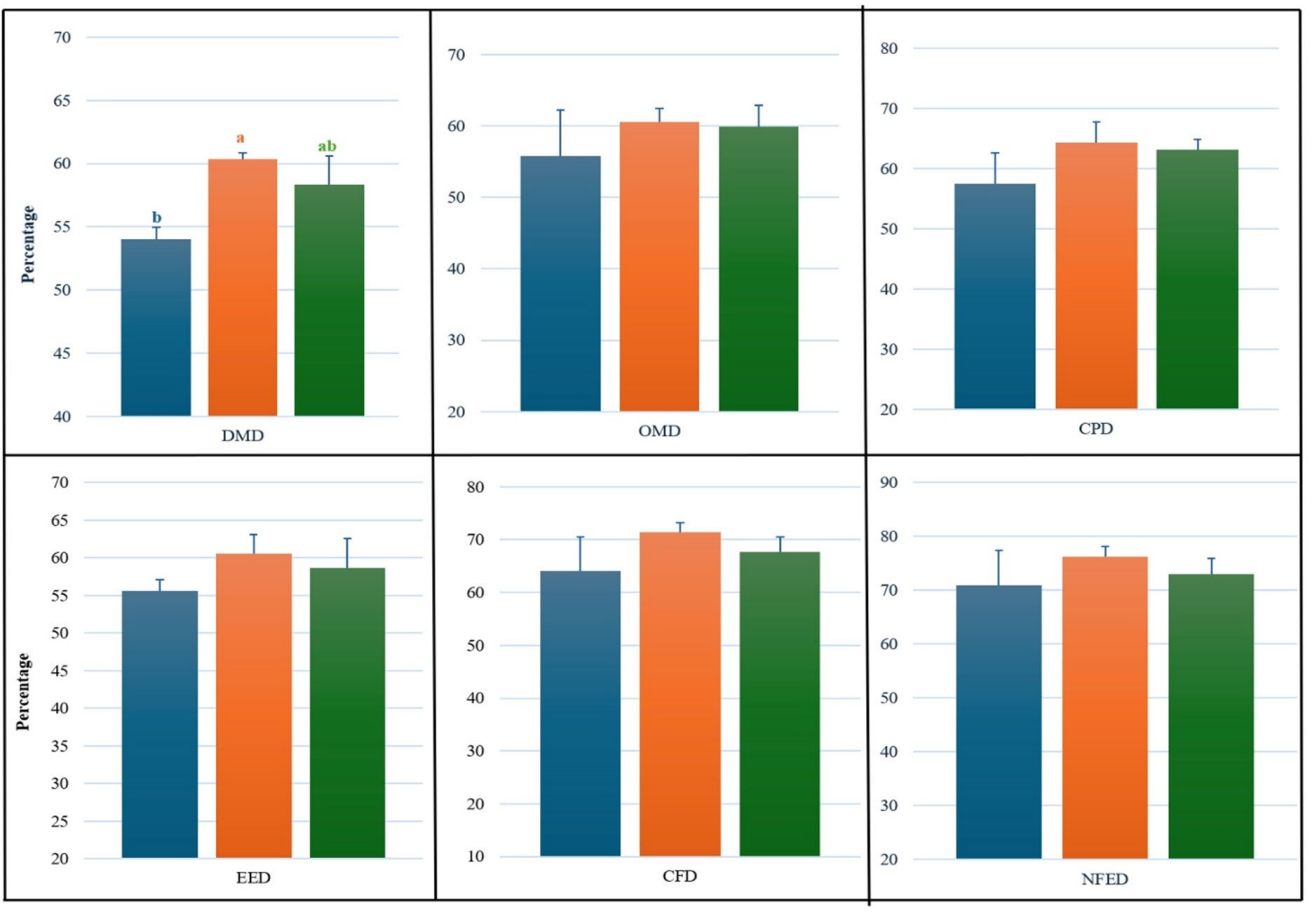
Apparent digestibility of nutrients in calves supplemented with LFM. Abbreviations: dry matter digestibility (DMD), organic matter digestibility (OMD), crude protein digestibility (CPD), ether extract digestibility (EED), crude fibre digestibility (CFD), and nitrogen-free extract digestibility (NFED). Letters (a, b) indicate *P* < 0.05. *n* = 5 calves per group.

Fermentation characteristics (Fig. 2) showed complementary shifts. At 2% inclusion, total volatile fatty acids (VFA) increased by 45.5% (*P* = 0.027) and total nitrogen (TN) by 34.1% (*P* = 0.025). Ammonia-nitrogen (NH₃–N) decreased by 28.4% at 2% inclusion and by 11.8% at 3% inclusion (treatment effect *P* = 0.018). Rumen pH (*P* = 0.106), soluble nitrogen (SN; *P* = 0.906), non-protein nitrogen (NPN; *P* = 0.652), and trichloroacetic-acid–soluble nitrogen (TCA-N; *P* = 0.956) were unaffected. These fermentation responses carried through into nutrient utilisation in vivo. Digestibility results are presented in Fig. 3. Dry matter digestibility (DMD) increased significantly at 2% and 3% inclusion (*P* < 0.05). Digestibility of organic matter (OM), crude protein (CP), ether extract (EE), crude fibre (CF), and nitrogen-free extract (NFE) did not differ significantly (*P* > 0.05), although numerical increases were observed. Methane emissions estimated from predictive equations (Fig. S1) followed the same trend, with reductions of 25.2% and 30.4% at 2% and 3% inclusion. Estimates from the IPCC (2006) Tier 2 model indicated comparable reductions of 19.3% and 19.2% (Table S1), demonstrating consistency across methods.

### Nitrogen dynamics

**Fig. 4:**
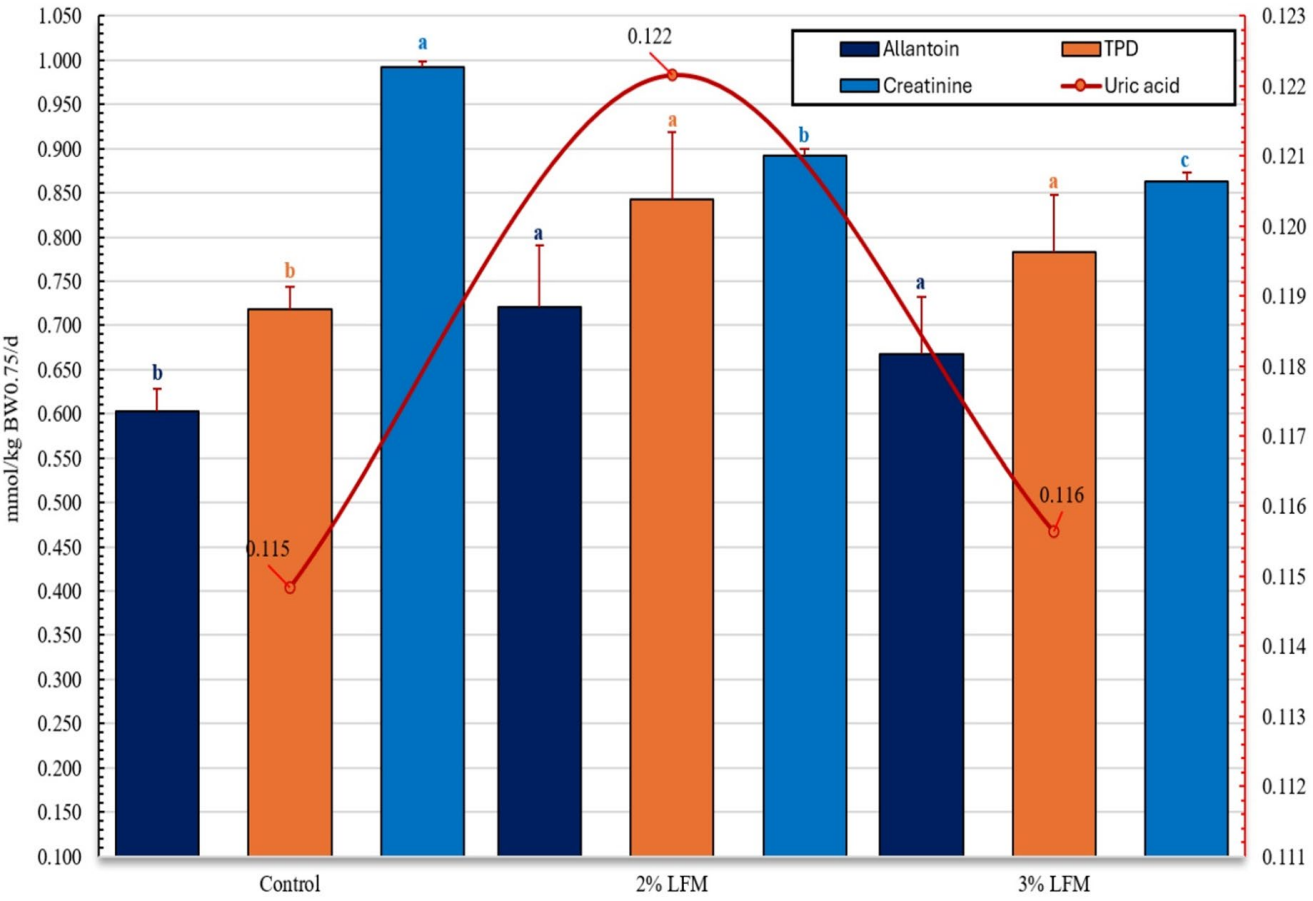
Metabolic biomarkers in calves supplemented with LFM. Outcomes include allantoin, uric acid, total purine derivatives (PD), and the PD:creatinine ratio. Bars show mean ± s.e.m. Letters (a–c) indicate *P* < 0.05. *n* = 5 per group.

Nitrogen metabolism, shown in Fig. 4, was also affected. Digestible crude protein (DCP) intake increased significantly across all measures (by 10–17%; *P* < 0.05). Calves supplemented with 2% and 3% LFM excreted more allantoin, total purine derivatives (PD), and had higher PD:creatinine ratios than controls (*P* < 0.05). Uric acid excretion did not differ (*P* > 0.05). Improvements in nitrogen use were accompanied by enhanced growth performance.

### Growth performance and efficiencies

Growth performance outcomes (Table 1) indicated that calves receiving 2% LFM gained 24.5% more bodyweight than controls. Feed conversion ratio (FCR), protein conversion ratio (PCR), and energy conversion ratio (ECR) improved significantly at 2% and 3% inclusion (*P* < 0.05). These improvements in efficiency were paralleled by favourable changes in haematological profiles.

**Table 1:** Growth, nutrient efficiency, and economics in calves supplemented with LFM.

	Control	2% LFM	3% LFM
BWG (g d⁻¹)	482.44^b^ ± 57.55	617.34^a^ ± 29.23	600.74^a^ ± 66.06
DM Intake (g kg⁻¹ BW⁰·⁷⁵)	126.97^b^ ± 6.41	114.95^a^ ± 5.34	117.90^a^ ± 6.31
CP Intake (g kg⁻¹ BW⁰·⁷⁵)	13.80^a^ ± 0.69	12.49^b^ ± 0.58	12.81^b^ ± 0.69
DCP Intake (g kg⁻¹ BW⁰·⁷⁵)	8.00^b^ ± 0.75	9.03^a^ ± 0.63	9.11^a^ ± 0.33
TDN Intake (g kg⁻¹ BW⁰·⁷⁵)	69.14^a^ ± 3.49	62.59^c^ ± 2.91	64.20^b^ ± 3.44
FCR (kg feed kg⁻¹ gain)	9.94^a^ ± 0.40	7.28^b^ ± 0.19	7.69^b^ ± 0.40
PCR (kg CP kg⁻¹ gain)	1.07^a^ ± 0.04	0.81^b^ ± 0.0s3	0.86^b^ ± 0.04
ECR (kg TDN kg⁻¹ gain)	5.41^a^ ± 0.22	3.96^b^ ± 0.10	4.18^b^ ± 0.22
Cumulative TMR intake (kg head⁻¹)	504.70 ± 38.54	473.25 ± 24.99	476.76 ± 27.47
Total LFM cost (US$ head⁻¹)	0.00	1.53 ± 0.08	2.31 ± 0.13
Total feed cost (US$ head⁻¹)	105.49 ± 8.06	100.44 ± 5.31	101.96 ± 5.87
Daily feed cost (US$ head⁻¹ d⁻¹)	1.08 ± 0.08	1.02 ± 0.05	1.04 ± 0.06
Feed cost (US$ kg⁻¹ gain)	2.23 ± 0.15	1.66 ± 0.09	1.78 ± 0.17
Reduction vs. control (feed cost per kg gain, %)		−25.56%	−20.18%

*Notes*: Values are mean ± s.e.m. Within a row, means without a common superscript (*a*, *b*, *c*) differ at *P* < 0.05 (one-way ANOVA with Tukey or Games–Howell as appropriate). BW⁰·⁷⁵, metabolic bodyweight; BWG, bodyweight gain; DM, dry matter; CP, crude protein; DCP, digestible crude protein; TDN, total digestible nutrients; FCR, feed conversion ratio; PCR, protein conversion ratio; ECR, energy conversion ratio; TMR, total mixed ration. Lower FCR/PCR/ECR values indicate greater efficiency. Currency values are presented in US dollars.

### Haematological profile

**Fig. 5:**
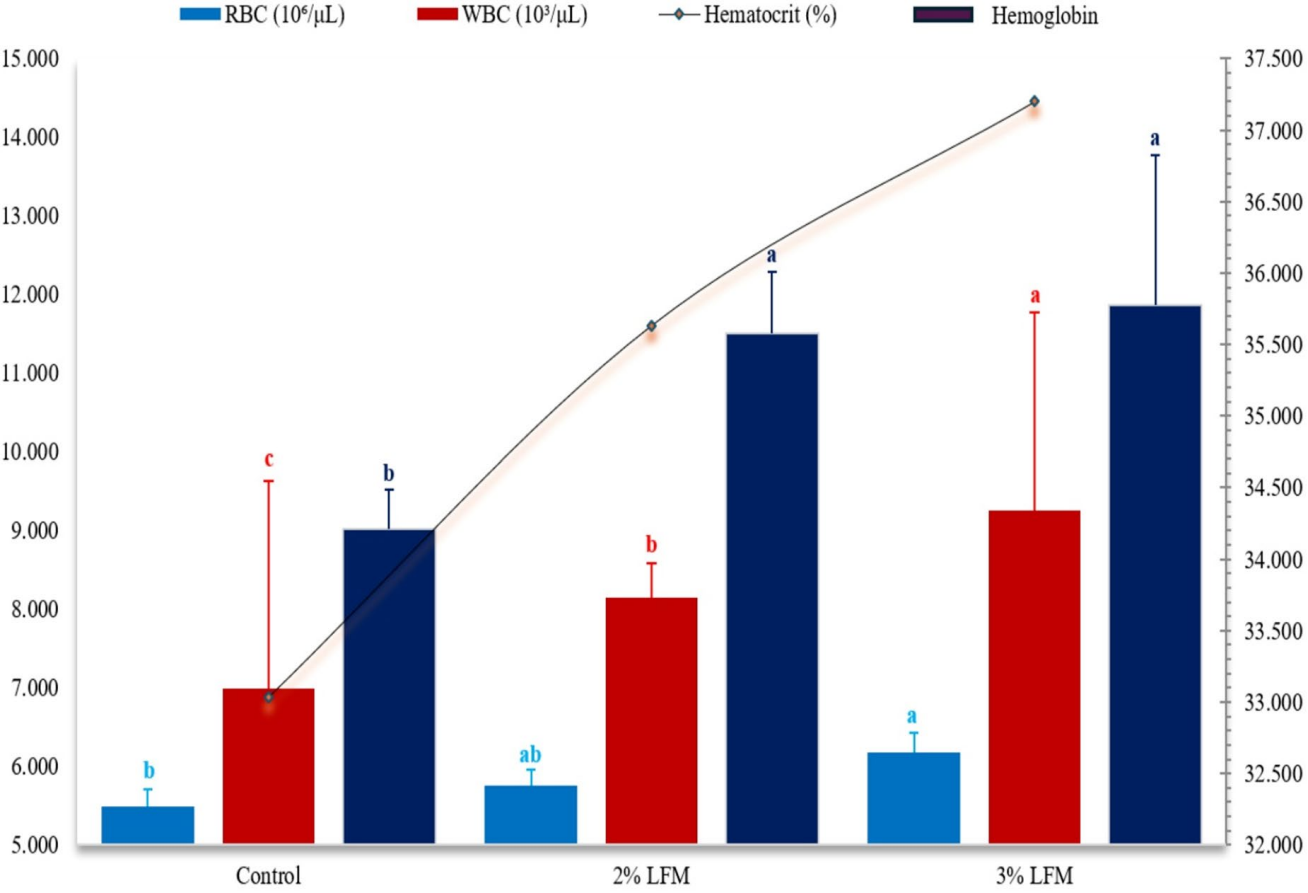
Haematological indices in calves supplemented with LFM. Hb (g dL⁻¹), RBC (10⁶ µL⁻¹), HCT (%), and WBC (10³ µL⁻¹). Bars show mean ± s.e.m. Letters (a–d) indicate *P* < 0.05. *n* = 5 per group.

Haematological results (Fig. 5) showed higher values at 3% inclusion. Red blood cell (RBC) counts (6.18 × 10⁶ cells µL⁻¹; ANOVA *F* = 4.279, *P* = 0.040), haemoglobin concentration (11.87 g dL⁻¹; *P* = 0.030), haematocrit (*P* = 0.035), and white blood cell (WBC) counts (9.26 × 10³ cells µL⁻¹; *P* = 0.004) were all elevated compared with controls. These systemic improvements aligned with enhanced growth and digestibility.

### In vitro- in vivo methane correlation and pan-India adoption scenario of LFM

**Fig. 6:**
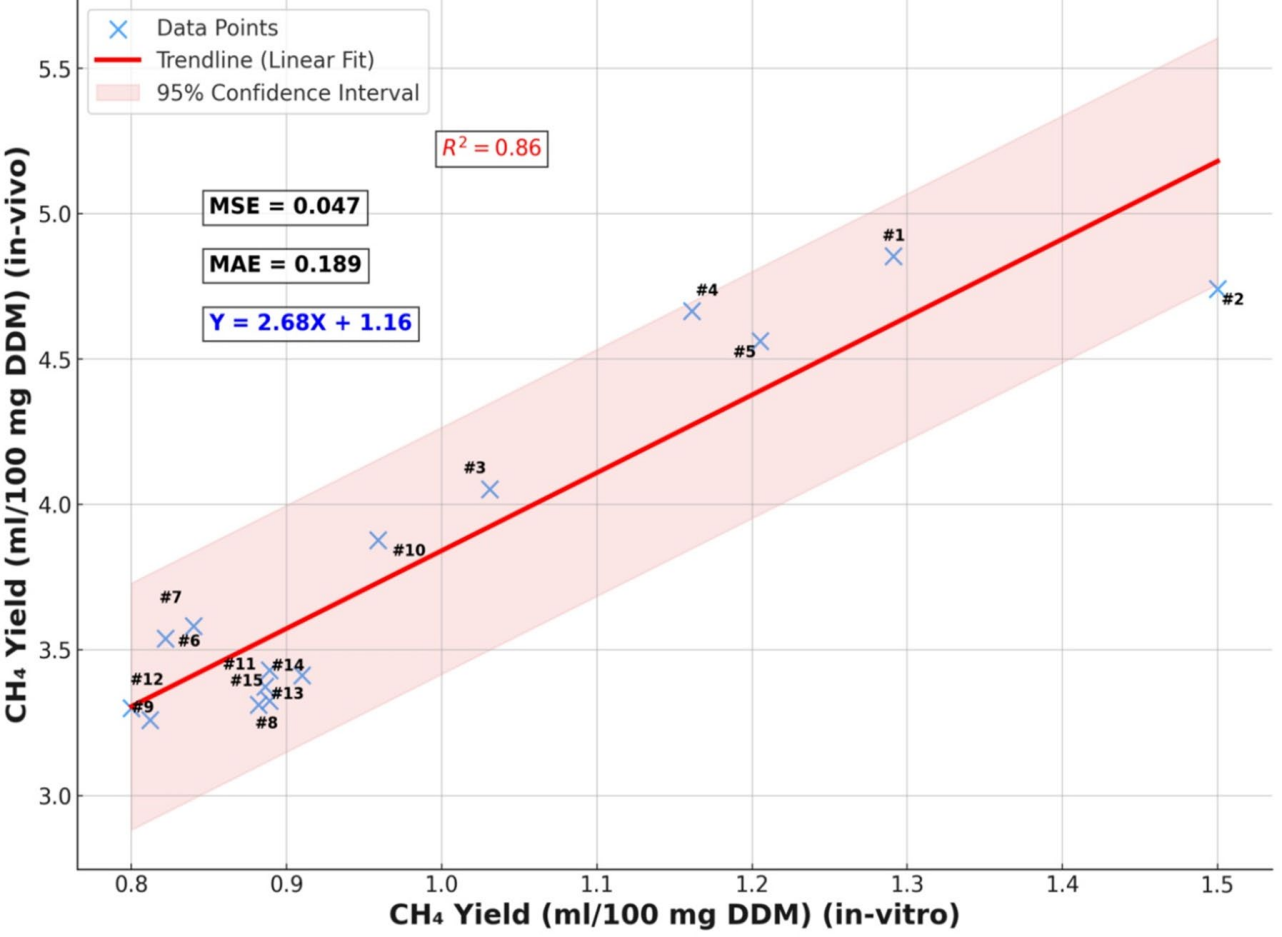
Relationship between in vitro and in vivo methane yields. Ordinary least-squares fit (R² = 0.86; Ŷ = 2.68X + 1.16).  The solid line shows the fitted relation; the shaded band indicates the 95% confidence interval.

**Fig. 7:**
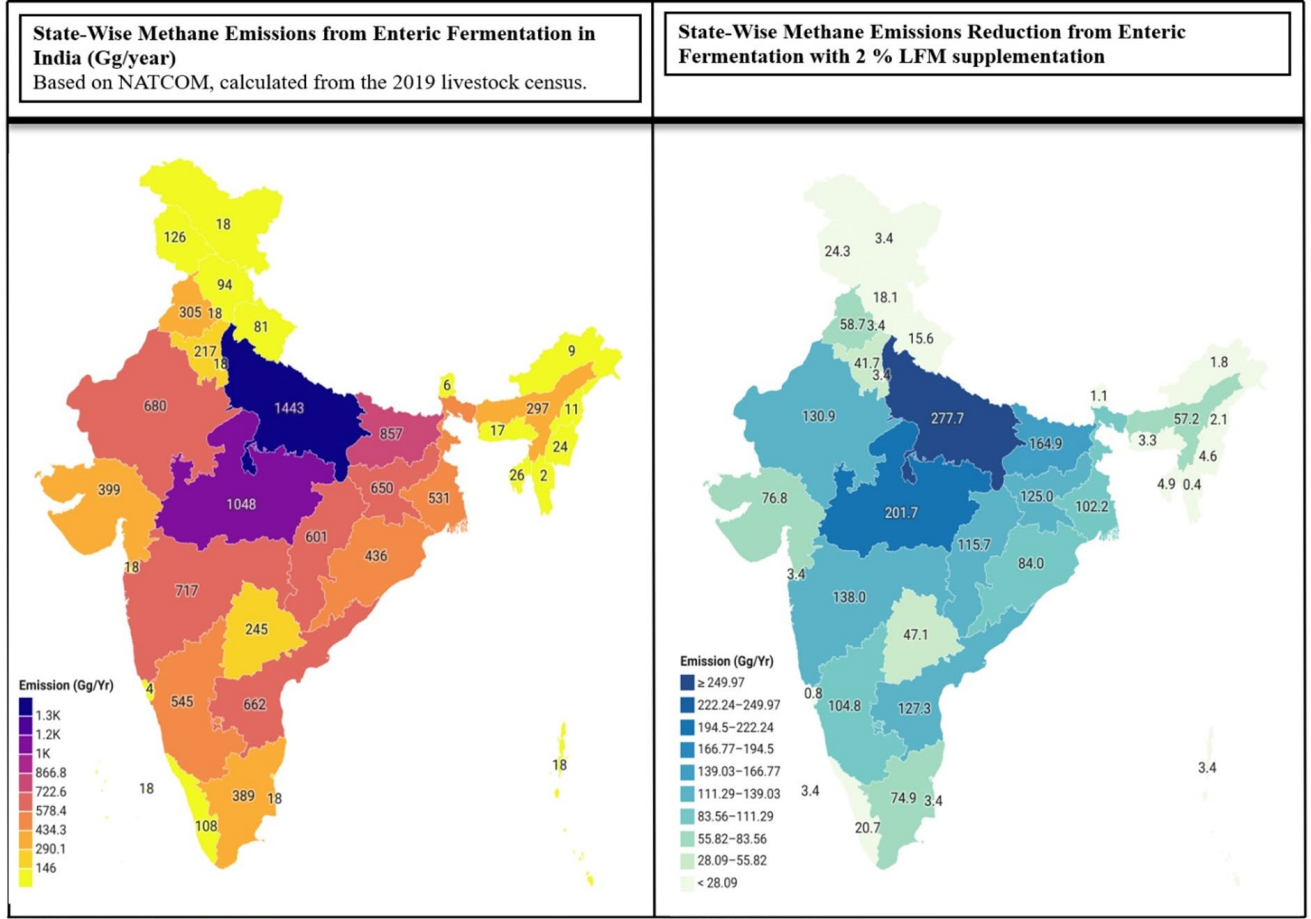
Spatial distribution of CH₄ emissions and mitigation potential with 2% LFM. Left: baseline enteric CH₄ emissions from Indian ruminants (Gg yr⁻¹), based on NATCOM data and the 2019 Livestock Census. Right: projected map showing a 20% reduction under 2% LFM inclusion, highlighting greater abatement potential in high-density states (e.g., Uttar Pradesh, Madhya Pradesh, Rajasthan).

In vitro and in vivo methane yields were strongly associated (Fig. 6). Linear regression analysis (*R²* = 0.86) yielded the equation Y (in vivo) = 2.68 × X (in vitro) + 1.16, with a mean squared error (MSE) of 0.047 and a mean absolute error (MAE) of 0.189. This demonstrated that in vitro assays reliably reflected in vivo outcomes within the tested range. National adoption scenarios are illustrated in Fig. 7 and Table S2. Extrapolation of the in vivo results suggested methane abatement of approximately 3.9 Mt CH₄ yr⁻¹ at 25% adoption and 15.4 Mt CH₄ yr⁻¹ under full adoption, equivalent to 432.3 Mt CO₂-eq yr⁻¹ (GWP₁₀₀ = 28). Carbon-credit revenues were estimated at US$494.1 million annually under full adoption and US$123.5 million at 25%. High livestock-density states such as Uttar Pradesh, Madhya Pradesh, and Rajasthan would deliver the largest reductions; for example, Uttar Pradesh alone could cut 277.7 Gg CH₄ yr⁻¹ at 2% inclusion. Together, these findings provide quantitative evidence that LFM inclusion improved digestibility and growth while reducing methane emissions at both herd and national scales.

## Discussion

Organic food waste typically exhibits high biochemical oxygen demand (BOD) and, if unmanaged, contributes to oxygen depletion, leachate generation, and fugitive emissions. In this study, we repurposed fruit- and vegetable-derived biowaste into a multistrain live feed microbial (LFM), which showed concurrent reductions in enteric methane along with gains in performance. Multistrain LFMs may offer broader functional coverage than single-strain products by combining hydrogen-utilising, lactate-utilising, and fibrolytic taxa; in our experiment, the formulation reduced methane without compromising nutrient digestibility, with higher total volatile fatty acids, increased purine-derivative:creatinine ratios, and lower NH₃–N, findings consistent with enhanced microbial nitrogen capture and a shift of reducing equivalents toward alternative sinks.

According to IPCC AR6, ruminant enteric methane emissions is the largest agricultural source of CH₄ and a major mitigation target^31^. Reducing these emissions is integral to meeting global climate objectives, including the Paris Agreement^32,33^. In India, livestock-related methane emissions are amplified by high ruminant densities, feed-quality constraints, and the traditional husbandry practices, with systems deeply embedded in rural economies^34–36^. Emerging microbial feed additives aim to redirect rumen hydrogen from methanogenesis to alternative sinks, lowering CH₄ while improving efficiency^37^, although adoption remains constrained by economic and logistical factors^38,39^.

Hydrogenotrophic methanogens dominate H₂ utilisation in the rumen, reducing CO₂ to CH₄ via methyl-coenzyme M reductase (MCR)^40–44^. This pathway favours elevated H₂ partial pressures on thermodynamic grounds^45,46^. Within the LFM, hydrogen redirection is plausibly supported by *Megasphaera elsdenii*, a lactate utiliser that channels reducing equivalents to propionate predominantly via the acrylate pathway in most strains, consistent with prior reports^47,48^. Mechanistically, propionate can arise either via the acrylate route (lactate → acrylyl-CoA → propionate) or via the succinate route (oxaloacetate → succinate → propionate); both rely on reduced cofactors (for example, NADH/FADH₂), but the acrylate route is typical for *M. elsdenii*^49,50^. Our observed CH₄ reductions and maintained digestibility are consistent with greater flow of reducing equivalents to propionate; we did not, however, quantify intermediates, so the pathway remains inferential. Greater propionate supply can also support hepatic gluconeogenesis and energy retention, with potential productivity benefits^51^.

In addition to *M. elsdenii*, *Clostridium butyricum* contributes to a more balanced VFA profile. Through butyryl-CoA dehydrogenase/electron-transferring flavoprotein complexes (flavin-based electron bifurcation), *C. butyricum* channels reducing equivalents toward butyrate, which can secondarily constrain H₂ availability for methanogenesis^52^. *Saccharomyces boulardii* (a *S. cerevisiae* variant used in humans) exhibits thermo- and acid-tolerance that can aid rumen survival; it differs from *S. cerevisiae* in its secreted enzyme profiles (for example, proteases and glycoside hydrolases)^53–57^, which may enhance fermentation efficiency and nutrient availability. Lactic acid bacteria—*Lactobacillus acidophilus*, *Lacticaseibacillus rhamnosus*, and *Lacticaseibacillus paracasei*—produce exopolysaccharides (EPS) that can stabilise microbial consortia, potentially facilitating biofilm formation and the adhesion of beneficial microbes. Proteolytic systems (endo- and aminopeptidases) may increase peptide and free-amino-acid availability, support microbial protein synthesis, and align with the lower NH₃–N observed here^58^. Their metabolism may interact with biohydrogenation, modulating unsaturated fatty acid conversion and generating conjugated linoleic acid (CLA) intermediates with potential host benefits^59^. Similarly, *Pediococcus acidilactici* may enhance competitiveness through bacteriocin production, constraint opportunistic taxa, and stabilise community structure^60^, while *Limosilactobacillus mucosae* may support biofilm formation via EPS synthesis, strengthening adhesion and resilience under fluctuating rumen conditions^61^. Finally, *Bifidobacterium bifidum* can hydrolyse oligosaccharides to fermentable sugars and support cross-feeding with hydrogenotrophs, which could secondarily reduce methanogenesis^37^.

LFM promotes the incorporation of ammonia into microbial biomass, supporting pathways of reductive amination and transamination^62,63^. These processes redirect nitrogen flux within the rumen, limiting NH₃–N accumulation and improving nitrogen cycling, which can reduce downstream nitrogen losses^64,65^.

In LFM-fed calves, higher excretion of purine derivatives (PD) indicates greater microbial nitrogen flow, plausibly aided by *Megasphaera elsdenii* diverting lactate towards propionate and thereby increasing the energy supply for NH₃–N assimilation^66^. In parallel, energy yield from fermentative metabolism in *Clostridium butyricum* may support microbial proliferation under nitrogen constraints^67,68^. Spores of *Bacillus coagulans* can persist under ruminal conditions and produce proteases that hydrolyse complex dietary proteins to simpler substrates, facilitating microbial protein synthesis^69^. This interpretation aligns with the observed rise in PD, a recognised proxy for microbial turnover and protein synthesis^70–72^.

Substantial increases in total volatile fatty acids (VFA) and total nitrogen (TN), together with stable ruminal pH and unchanged nitrogen pools [soluble nitrogen (SN), non-protein nitrogen (NPN), and trichloroacetic-acid–soluble nitrogen (TCA-N)], suggest a resilient microbial ecosystem. The buffering capacity of *Saccharomyces boulardii* may help limit acid build-up and maintain community stability^73,74^.

By shifting nitrogen from ammonia towards microbial biomass, LFM supplementation may reduce ammonia volatilisation and minimise post-ruminal nitrogen losses, increasing the availability of metabolisable nitrogen. These patterns are consistent with improved feed conversion efficiency (FCE) and bodyweight gain (BWG), reducing reliance on external protein sources. Collectively, the benefits align with Sustainable Development Goals, particularly SDG 13 (Climate Action) and SDG 12 (Responsible Consumption and Production), and with prior work on the economic value of nitrogen-efficient feeding strategies^75,76^.

Repurposing food-chain residues into live microbial feed additives operationalises the circular economy by diverting organic wastes from disposal and substituting finite inputs in feed production. In our scaling analysis, full national adoption of LFM could abate 15.44 Mt CH₄ yr⁻¹, equivalent to 432.32 Mt CO₂-eq yr⁻¹ using GWP₁₀₀ = 28^77–79^.

India’s ~68 Mt yr⁻¹ food-chain surplus/waste^80,81^ provides a practical feedstock base for such bioprocessing. With local calibration of strains, diets, and delivery formats, the same model could be adapted to other high-emission livestock regions, including Brazil and selected systems in sub-Saharan Africa^82,83^. This pathway strengthens material and nutrient circularity by converting biowaste into productive inputs and by reducing methane and reactive-nitrogen losses along the agri-food chain.

Alignment with international commitments is direct. Enteric-methane abatement contributes to targets under the Paris Agreement and the Global Methane Pledge^84–86^. Where measurement, reporting and verification (MRV) systems are robust, quantified reductions may be eligible for carbon markets; any revenue depends on crediting standards, baselines, additionality, leakage, permanence, and prevailing prices^87–90^.

Given methane’s short atmospheric lifetime (~ 12 years) and high near-term warming impact (GWP₂₀ ≈84; GWP₁₀₀ ≈ 28), accelerating CH₄ abatement yields outsized climate benefits^91^. LFM offers a hydrogen-repartitioning, circular-economy intervention that can complement diet formulation, genetics, and manure management within national methane-reduction portfolios, subject to context-specific life-cycle, safety, and cost–benefit evaluations.

## Methods

### Ethics statement

All procedures were approved by the Institutional Animal Ethics Committee (IAEC), Kamdhenu University (protocol IAEC 314/ANRS/2020). The study complied with the Animal Research: Reporting of In Vivo Experiments (ARRIVE) guidelines, and all methods were carried out in accordance with relevant guidelines and regulations. Calves were sourced from the Livestock Research Station (LRS), Kamdhenu University, Anand, India.

### Study design overview

The work comprised three phases: (1) development of the live feed microbial (LFM); (2) in vitro determination of dry-matter digestibility (IVDMD) and in vitro methane production (IVM); and (3) an in vivo digestibility and performance trial.

### Development of LFM

A total of ten microbial strains—*Lacticaseibacillus paracasei* (syn. *Lactobacillus paracasei*), *Lactobacillus acidophilus*, *Lacticaseibacillus rhamnosus* (syn. *Lactobacillus rhamnosus*), *Weizmannia coagulans* (syn. *Bacillus coagulans*), *Megasphaera elsdenii*, *Pediococcus acidilactici*, *Clostridium butyricum*, *Limosilactobacillus mucosae*, *Bifidobacterium bifidum,* and *Saccharomyces boulardii*—were isolated from rumen fluid and raw milk collected from healthy dairy cows maintained under controlled husbandry conditions at the Animal Nutrition Research Station Farm, Kamdhenu University, Anand, Gujarat, India. Strain identification and characterisation used Biolog GEN III microplates (Biolog, Hayward, CA, USA) and MALDI-TOF MS (e.g., Bruker Biotyper; species-level score thresholds applied per manufacturer guidance). Candidate probiotics were prioritised for their capacity to metabolise lactic acid to short-chain fatty acids (SCFA; acetate, propionate, butyrate, valerate), quantified by ion chromatography.

Each strain was grown separately in sterile liquid medium containing (per litre): peptone (10–15 g L⁻¹), beef extract (9–11 g L⁻¹), yeast extract (4–6 g L⁻¹), dextrose (15–20 g L⁻¹), polysorbate-80 (0.8–1.0 g L⁻¹), dipotassium hydrogen phosphate (K₂HPO₄; 1.5–2.0 g L⁻¹), magnesium sulphate (MgSO₄; 0.075–0.10 g L⁻¹), manganese(II) sulphate (MnSO₄; 0.040–0.060 g L⁻¹), and sodium acetate (0.10–0.12 g L⁻¹) in deionised water to a final volume 1.0 L. The medium pH was adjusted to 6.4–6.6 using 1 M HCl or NaOH and sterilised at 121 °C for 15 min (≈ 103 kPa). Cultures were incubated at 37 ± 2 °C for 48–56 h until reaching approximately 10⁶ CFU mL⁻¹. For the inoculum, strains were pooled after CFU normalisation to ensure equal contribution to the final mixture.

Fruit- and vegetable-market waste was dried, milled, and sterilised, then inoculated with the pooled culture to an initial load of 10⁴–10⁵ CFU g⁻¹. Solid-state fermentation proceeded in tray fermenters at 35 ± 1 °C and 85–90% relative humidity for 72–96 h, yielding a fermented biomass containing measurable SCFA and viable counts exceeding 10⁷–10⁸ CFU g⁻¹. The fermented product was stabilised by blending with a sterile dry carrier to reduce moisture to < 15%, combined with a binder, and then cold-pressed into consumable forms. Final products were vacuum-sealed for shelf stability and microbial viability. The full development protocol is detailed in Indian Patent Application No. 202,421,030,268.

### Determination of IVDMD

To identify the optimal inclusion level of LFM in a jowar (sorghum) straw–based total mixed ration (TMR) (Fig. S2), an in vitro digestibility assay was conducted. Rumen liquor was collected from calves after a 24-h fast via aseptic oesophageal intubation and filtered through muslin to remove particulates. The strained rumen liquor (SRL) was transferred to pre-warmed, CO₂-flushed thermos flasks to maintain anaerobiosis at 39 ± 1 °C. The McDougall’s buffer used in the assay is detailed in Table S3.

TMR samples were ground through a 1.0-mm screen, supplemented with LFM at 1–7% of dietary dry matter (DM), and loaded in triplicate into 100-mL glass syringes. Macro- and micro-mineral solutions were prepared and pre-equilibrated at 39 °C^92^. For each syringe, 500 mg of TMR was weighed on an analytical balance, SRL was combined with McDougall’s buffer under continuous CO₂ flushing, and the inoculated syringes were sealed with rubber stoppers. Syringes were incubated horizontally in a shaking water bath at 39 ± 1 °C for 24 h, with gentle agitation every 4 h to mimic rumen motility and ensure thorough mixing^93,94^.

After incubation, contents were filtered through pre-weighed Whatman No. 54 filter papers. Residues were washed with deionised water, oven-dried at 105 °C to constant mass (≤ 24 h), cooled in a desiccator, and re-weighed. In vitro dry-matter digestibility (IVDMD) was calculated as: IVDMD (%) = [(Initial dry weight − Residual dry weight)/Initial dry weight] × 100.

### Quantification of IVM

#### Substrate Preparation

Substrates (200 mg) were ground through a 1.0 mm screen to standardise particle size and microbial accessibility. Milled substrates were stored in hermetically sealed containers at 4°C to limit moisture uptake and pre-incubation degradation.

### Incubation conditions

Samples were incubated at 39 ± 1 °C in a dual-chamber shaking water bath following Menke’s gas-production protocol^95^ (see Table S3), ensuring uniform mixing and temperature control. Bath temperature was verified before each run using a NIST-traceable thermometer. Each substrate was incubated for 48 h with 40 mL of artificial-saliva buffer inoculated with strained rumen liquor (SRL). All substrate, LFM, and SRL handling occurred in an anaerobic chamber (95% N₂/5% H₂) to maintain anaerobiosis. Incubation bottles were sealed with butyl-rubber stoppers and aluminium crimp caps. Headspace O₂ was monitored with a calibrated sensor and maintained at < 0.1%.

### Gas measurement and calculations

Total gas volume was recorded at the end of incubation following Menke^95^. Headspace CH₄ fraction was determined by gas analysis as detailed in Table S4, using certified standards; blanks (buffer + SRL without substrate) were run in parallel^96,98^. Methane volume (mL) was calculated as total gas × CH₄ fraction, blank-corrected, and expressed per unit of incubated dry matter (DM) and per 100 mg of digestible dry matter (DDM): IVM (mL 100 mg⁻¹ DDM) = [CH₄ (mL) ÷ DDM (mg)] × 100.

### Gas chromatography analysis

Headspace gas was withdrawn from incubation vessels using 500 µL gas-tight syringes (manual injection); syringes were flushed with N₂ between samples to minimise carry-over, and duplicate injections were performed for each vial. Methane was quantified by gas chromatography using a packed stainless-steel column (Porapak N, 80/100 mesh; 4 ft × 3.2 mm i.d.) coupled to a flame-ionisation detector. The oven was held isothermal at 50 °C; carrier gas was N₂ (99.999%) at 30 mL min⁻¹ under mass-flow control. Injector and detector temperatures were set at 150 °C and 200 °C, respectively, with standard FID fuel and oxidant flows. Data were acquired in Agilent ChemStation, and CH₄ fractions were derived from integrated peak areas using external calibration with certified, NIST-traceable standards (10.4 and 101.9 ppmv; Scott-Marrin, USA), which yielded linear responses (R² > 0.999). Gas cylinders supplied a two-stage regulator and high-purity distribution manifold to avoid contamination; instrument blanks and headspace blanks (buffer + SRL) accompanied each batch, and replicate injections required < 5% RSD. Sample CH₄ volume was calculated as total gas volume multiplied by the calibrated CH₄ fraction, blank-corrected, and expressed per unit of incubated dry matter (DM) and per 100 mg of digestible dry matter (DDM) to align with IVM reporting. Percentage reduction versus control was computed as 100 × [1 − (CH₄_treatment/CH₄_control)].

### In vivo animal trials and quantitative rumen microbial dynamics

#### Experimental animals, design, treatments, and feeding

The trial included a 14-day adaptation followed by a 98-day treatment phase. Fifteen indigenous Kankrej calves (6–12 months old) were enrolled and randomly allocated (*n* = 5 per group) to three dietary treatments: control (no LFM), 2% LFM, and 3% LFM (percentage of diet dry matter; Table S5). All animals were offered a total mixed ration (TMR) formulated according to Indian Council of Agricultural Research (ICAR) recommendations. The basal TMR contained ~11% crude protein (CP) and 54.45% total digestible nutrients (TDN) (Table S6) and was fed at a roughage:concentrate (R:C) ratio of 50:50 (Table S7), a proportion selected on the based on prior calorimetric work indicating stable fermentation and lower methane at 50:50–58:42 R:C^12,15^.

### Rumen fermentation dynamics

Rumen liquor was collected using sterile oesophageal stomach-tubing 3 h post-feeding to coincide with peak microbial activity. Samples were transferred immediately into pre-chilled airtight tubes, placed on ice, and transported to the laboratory within 10 min while minimising oxygen exposure. In the laboratory, contents were filtered through sterile muslin to remove coarse particles, and rumen pH was recorded in duplicate on fresh aliquots.

### Nitrogen fractions analysis

NH₃–N was determined colorimetrically by the indophenol (phenate) method of Pearson and Smith^97^. SRL was filtered through Whatman No. 1 filter paper, handled in low-nitrogen glassware, and analysed within 2 h; If analysis was delayed, aliquots were acidified to pH < 2 with H₂SO₄ and stored at 4 °C. For each assay, 5.0 mL SRL was mixed with 1.0 mL phenol solution (50 g L⁻¹), 0.5 mL sodium nitroprusside (0.5 g L⁻¹), and 1.0 mL alkaline sodium hypochlorite (5% w/v active chlorine), incubated at 37 °C for 20 min, and read at 630 nm (10 mm cuvette) against reagent blanks. Concentrations were obtained from matrix-matched ammonium-sulfate standards spanning the sample range (linear fit, R² > 0.999) and reported as mg NH₃–N L⁻¹. Duplicate determinations were required to agree within 5% RSD; spike-recovery checks (90–110%) were included per batch to confirm the absence of matrix interference.

TN was measured by Kjeldahl digestion. SRL aliquots were digested in concentrated H₂SO₄ with a mixed catalyst, made alkaline, steam-distilled, and the evolved ammonia was trapped in boric acid and titrated with standardised HCl; results were expressed as mg N L⁻¹. Soluble nitrogen (SN) was measured in clarified supernatant (3,000 × g, 15 min, 4 °C) using the same Kjeldahl workflow. Non-protein nitrogen (NPN; TCA-soluble N) was obtained by adding trichloroacetic acid to 5% (w/v), holding samples on ice to precipitate proteins, centrifuging as above, and analysing the supernatant by Kjeldahl. Blanks and a mid-range quality-control standard accompanied each run.

VFA were determined by steam distillation using a Markham micro-distillation apparatus. Fresh SRL for VFA analysis was preserved immediately (pH < 2 with H₂SO₄) and processed on the same day. Distillate was collected into 0.1 M NaOH and back-titrated with standardised HCl to the phenolphthalein endpoint (pH ≈ 8.3). After blank correction, total VFA was calculated and expressed as mmol L⁻¹.

### Spot sampling technique for estimating purine derivative (PD)

Spot urine (~100 mL) was collected at voiding into sterile containers 3 h post-feeding on two non-consecutive days per calf, mixed, aliquoted, and frozen at −20 °C. To prevent degradation of purines, an aliquot from each sample was acidified at collection with H₂SO₄ to pH < 3. Before analysis, samples were thawed at 4 °C, remixed, adjusted to assay pH, and diluted with deionised water to ensure all readings within the linear range of each assay.

Allantoin was quantified by the phenylhydrazine/glyoxylate reaction (≈522 nm). Uric acid was quantified enzymatically (uricase–peroxidase, ≈520–540 nm). Creatinine was measured enzymatically (creatininase) to avoid Jaffé interference. Assays used 1 cm cuvettes and five-point calibrations with reagent blanks; duplicate readings were accepted at < 5% RSD, spike-recovery was 90–110%, and results were verified to fall within calibration range and LOQ. Creatinine concentrations were expressed in mmol L⁻¹; when instruments output mg L⁻¹, values were converted using a molecular weight of 113.12 mg mmol⁻¹.

PD concentration was calculated as PDconc = allantoin + uric acid (mmol L⁻¹). Xanthine and hypoxanthine were negligible under the assay conditions. Because only spot urine was collected, daily urine volume (L d⁻¹) was estimated from creatinine dilution using a herd-specific creatinine-excretion constant on a metabolic-weight basis (kcr = 0.98 mmol kg⁻⁰·⁷⁵ d⁻¹): Volume (L d⁻¹) = [kcr × BW⁰·⁷⁵ (kg⁰·⁷⁵)]/UCconc (mmol L⁻¹), where BW is body weight and UCconc is urinary creatinine concentration. Daily PD excretion was then: Y (mmol d⁻¹) = PDconc (mmol L⁻¹) × Volume (L d⁻¹). When daily urine volume could not be estimated (e.g., out-of-range creatinine), the PD:creatinine ratio (mmol mmol⁻¹) was used as a dilution-normalised index^99,100^.

Absorbed purines were derived from urinary PD using the standard cattle relationship: Y (mmol d⁻¹) = 0.85 × X (mmol d⁻¹) + 0.385 × BW⁰·⁷⁵ (mmol d⁻¹), where 0.85 is the average urinary recovery of absorbed purines and 0.385 mmol kg⁻⁰·⁷⁵ d⁻¹ is the endogenous PD term. Solving for absorbed purines: X (mmol d⁻¹) = [Y − 0.385 × BW⁰·⁷⁵]/0.85^101^.

### Haematological analysis

Blood was drawn by jugular venepuncture each morning with calves standing and minimally restrained, without venous occlusion. Whole blood was collected into K₂-EDTA vacutainers (final EDTA 1.5–2.0 mg mL⁻¹), mixed gently (8–10 inversions), and inspected for clots, haemolysis, or lipaemia; flagged samples were re-drawn. Complete blood counts were run within 2 h of collection and samples were kept cool until analysis. For ancillary assays compatible with EDTA, plasma was prepared at 1,500 × g for 15 min at 4 °C, aliquoted, and stored at −20 °C (single freeze–thaw cycle maximum).

Haemoglobin (Hb), red blood cell (RBC) count, white blood cell (WBC) count, and haematocrit (HCT) were measured from fresh EDTA blood on an automated veterinary haematology analyser (BC-2800 Vet, Mindray; bovine mode) following the manufacturer’s instructions. Instrument performance was verified daily with commercial controls, and analytical runs were accepted only when control values fell within target ranges. Samples flagged by the instrument flags re-analysed, and a Wright–Giemsa-stained blood smears were examined when indicated.

Packed cell volume (microhaematocrit) was measured as an orthogonal check by loading EDTA blood into plain capillary tubes, sealing, and centrifuging at ~12,000 × g for 5 min; readings (excluding the buffy coat) were taken with a standard haematocrit reader. If analyser HCT and microhaematocrit differed by > 3% points or > 5% relative, the sample was re-run and the smear re-examined. Sampling time, operator, and handling were standardised across groups to minimise diurnal and pre-analytical variation.

### In vivo methane production Estimation

Enteric CH₄ production was estimated using two complementary approaches. First, an Ellis^102^ model was applied using each calf’s organic matter intake (OMI) and digestible organic matter (DOM), derived from recorded intakes and in vivo digestibility measurements. Outputs were expressed as CH₄ production (g d⁻¹) and as CH₄ yield (g kg⁻¹ DMI). Second, the IPCC (2006) Tier 2 methodology was applied.

To account for the multifactorial nature of methane emissions, additional predictive models^103–107^ were integrated. These models consider key variables such as dry matter intake (DMI), gross energy intake (GEI), and organic matter intake (OMI), complementing the digestion- and energy-based estimates generated by the Ellis and IPCC models. A summary of the methane emission estimates derived from these models is presented in Table S5.

### National extrapolation model for CH₄ reduction

We scaled animal-level effects to the national herd using headcounts from India’s 20th Livestock Census (stratified by state, species, and production class) and the IPCC (2006) Tier 2 baseline. For each class, gross energy intake (GEI; MJ d⁻¹) was calculated from dry-matter intake and diet gross-energy density. Daily enteric methane production was estimated as CH₄ (kg d⁻¹) = [GEI × Yₘ/100] ÷ 55.65, where Yₘ is the methane conversion factor (set to 5.5% for post-weaned, forage-based systems) and 55.65 MJ kg⁻¹ represents the energy content of methane. Annual per-head emissions were derived by multiplying daily CH₄ by 365; state-level values were then summed to a national baseline.

Abatement was modelled as a proportional reduction of this baseline using the in vivo effect size from the present study. The reduction factor r was defined as the Tier-2-consistent proportional decrease in methane at the specified LFM inclusion level (2% or 3%) and was applied uniformly to eligible cattle and buffalo unless otherwise stated. Adoption scenarios of 25%, 50%, 75%, and 100% were modelled by multiplying the baseline by r and the assumed adoption fraction (national abatement = baseline × r × adoption fraction). Where required for comparison with nutrition metrics, outcomes were also expressed as CH₄ yield (g kg⁻¹ DMI) using the same intake parameters.

Methane reductions were converted to CO₂-equivalent (CO₂-eq) using GWP₁₀₀ = 28 to maintain consistency across the manuscript. Potential carbon-credit values were treated parametrically as CO₂-eq (t yr⁻¹) multiplied by an assumed unit price (US$ t⁻¹); price assumptions are reported with results rather than embedded in the methodology.

Uncertainty was assessed deterministically by varying Yₘ by ± 1% percentage point, GEI by ± 10%, and r across the range indicated by intake-based prediction equations. Only enteric CH₄ was quantified (manure and other life-cycle emissions were excluded to avoid double counting). Adoption was treated as a scenario, not a forecast. All calculations used consistent units and the same intake and digestibility datasets as those used in the animal trial.

### Statistical analysis

Analyses were performed in SPSS v29.0.2.0; Excel (MSO v2406) was used only for data handling/plots. The calf was considered the experimental unit (*n* = 5 per group). Technical replicates (e.g., triplicate syringes) were averaged per animal or per bottle before analysis. Results are reported as mean ± s.e.m.; all tests were two-sided with α = 0.05.

For in vivo endpoints (intake, digestibility, nitrogen fractions, haematology, growth indices, methane estimates), one-way ANOVA was applied with treatment as a fixed factor (control, 2% LFM, 3% LFM). Assumptions were checked on residuals: normality by Shapiro–Wilk and homogeneity of variance by Levene’s test. When variances were unequal, Welch’s ANOVA was used. Pairwise comparisons used Tukey’s HSD when variances were homogeneous and Games–Howell when heterogeneous.

In vitro dose-response series (1–7% LFM) were analysed by one-way ANOVA across doses. The association between in vitro and in vivo methane outputs was evaluated using ordinary least-squares regression; slope, intercept, R², mean-squared error (MSE), and mean-absolute error (MAE) are reported. No data imputation was performed; observations failing pre-specified quality-control criteria were excluded.

### Conclusions

Converting fruit- and vegetable-derived biowaste into a multistrain LFM yielded ~ 20–25% modelled reductions in enteric CH₄ and improved performance (higher DMD, better FCR, greater BWG) on a 50:50 roughage:concentrate diet. This links methane mitigation with waste valorisation using processes compatible with routine feed manufacture.

#### Future Directions

Since in vivo CH₄ was estimated from intake-based models (not chambers/SF₆/GreenFeed); group size was small (*n* = 5), single site/breed, two doses, one basal diet; economic outputs used average prices; product stability/handling were not stress-tested, it is advised to conduct a multi-site, adequately powered, randomised trial with direct CH₄ measurement (chambers for calibration; GreenFeed/SF₆ for throughput), a 0–4% dose–response across at least two diets, and preregistered primary endpoints (CH₄ g d⁻¹ and g kg⁻¹ DMI, FCR, BWG). Pair performance with targeted ¹⁵N tracing of microbial-N flow and focused pathway markers for hydrogen sinks (rather than broad descriptive ‘omics). Finalise translation components: lot-release specs (identity, contaminants, CFU g⁻¹ at end-of-shelf-life), real-time stability and simple field-handling tests, a cradle-to-farm-gate LCA with stated boundaries, and a Tier-2-aligned MRV protocol. Include AMR-gene screening and adverse-event monitoring.

## Supplementary Information

Below is the link to the electronic supplementary material.

Supplementary Material 1

## Data Availability

All the data generated or analysed during this study are included in this article and its supplementary information files.
