# Supplementary material for: Biowaste-grown live microbial feed additive sustainably and significantly cut enteric methane emissions in Indian livestock
Source: Sci Rep. 2026 Jan 13;16:1767. doi: 10.1038/s41598-025-29303-9 (PMC12804752; doi:10.1038/s41598-025-29303-9)
Supplement: Supplementary file 1 — Supplementary Material 1 [file 41598_2025_29303_MOESM1_ESM.docx]

**Table S1. Methane Emission Reduction Estimates Using Predictive Equations**

| Reference | Equations | Methane Reduction | |
| --- | --- | --- | --- |
|  |  | **2% LFM** | **3% LFM** |
| IPCC (2006) | CH₄ (kg/d) = (GE (MJ/d) × Ym) / 55.65 | 19.25 | 19.19 |
| Yan *et al*. (2009) | CH₄ (L/d) = 1.959 × GEI (MJ/d) + 8.8 | 17.47 | 17.40 |
| Grainger *et al*. (2007) | CH₄ (g/d) = 18.5 × DMI (kg/d) - 9.5 | 11.96 | 10.42 |
| Kennedy and Charmley (2012) | CH₄ (g/d) = 19.6 × DMI (kg/d) | 10.41 | 9.08 |
| Stergiadis *et al*. (2015) | CH₄ (g/d) = 50.85 + 21.63 × OMI (kg/d) | 6.18 | 5.40 |

*Abbreviations: GE = Gross Energy (MJ/day), Ym = Methane emission factor (MJ/100 MJ GE),

DMI = Dry Matter Intake (kg/day)

**Table S2. Estimated methane abatement and carbon credit potential from varying adoption levels of 2% LFM supplementation in Indian livestock populations**

| Adoption Rate | Livestock Population Involved (millions) | Total Methane Emissions (MT/year) | Methane Reduction with 2 LFM (MT/year) | CO_2_ equivalent Emissions Reduction (MT/year) * | Carbon Credits Earned (million USD) * |
| --- | --- | --- | --- | --- | --- |
| 25 % | 133.75 | 20.06 | 3.86 | 108.08 | 123.52 |
| 50 % | 267.50 | 40.12 | 7.72 | 216.16 | 247.04 |
| 75 % | 401.25 | 60.18 | 11.58 | 324.24 | 370.56 |
| 100 % | 535.00 | 80.25 | 15.44 | 432.32 | 494.08 |

*Based on an average methane emission factor of 150 kg CH₄ per animal per year, the projected reductions in methane emissions with LFM are presented in million tonnes (MT) per year. The CO₂-equivalent reductions are calculated using methane’s global warming potential (GWP) of 28.

**Table S3. Composition of McDougall buffer used for *in-vitro* studies.**

| Particulars | Quantity |
| --- | --- |
| Solution A (microminerals) for 100 ml | |
| Calcium chloride (CaCl_2_.2H_2_O) | 13.2 g |
| Manganese chloride (MnCl_2_.4H_2_O) | 10.0 g |
| Cobalt chloride (CoCl_2_.6H_2_O) | 1.0 g |
| Iron chloride (FeCl_2_.6H_2_O) | 8.0 g |
| Distilled water (final volume) | To make 100 ml |
| Solution B (buffer solution) | |
| Sodium hydrogen carbonate (NaHCO_3_) | 35.0 g |
| NH_4_ HCO_3_ (ammonium hydrogen carbonate) | 4.0 g |
| Distilled water (final volume) | To make 1000 ml |
| Solution C (macrominerals) | |
| Disodium hydrogen phosphate (Na_2_HPO_4_) | 5.7 g |
| Potassium dihydrogen phosphate  (KH_2_PO_4_) | 6.2 g |
| Magnesium sulphate (MgSO_4_.7H_2_O) | 0.6 g |
| Distilled water (final volume) | To make 1000 ml |
| Resazurin solution | |
| Resazurin | 100 mg |
| Distilled water (final volume) | To make 100 ml |
| Reducing solution | |
| Sodium hydroxide (1NaOH) | 4 ml |
| Sodium sulphide (Na_2_S.9H_2_O) | 625 mg |
| Distilled water (final volume) | To make 100 ml |

**Table S4. Media composition for in vitro gas production technique**

| Particulars | SRL and the quantity of various solutions for DMD |
| --- | --- |
| Solution A (Micro) (ml) | 0.12 |
| Solution B (buffer) (ml) | 237.78 |
| Solution C (Macro) (ml) | 237.78 |
| Resazurin solution (ml) | 1.19 |
| Reducing solution (ml) | 47.56 |
| Distilled water (ml) | 475.57 |
| Total media (ml) | 1000 |
| Rumen liquor (ml) | 500 |
| Total mixture (ml) | 1500 |

**Table S5. Experimental treatments and diets of animals**

| Treatments | Number of animals | Treatment diet |
| --- | --- | --- |
| Control | 5 | Control TMR (Roughage to Concentrate ratio of 50:50) |
| 2% LFM | 5 | Control TMR + LFM (1^st^ best *in-vitro* level) |
| 3% LFM | 5 | Control TMR + LFM (2^nd^ best *in-vitro* level) |

**Table S6. Nutrient composition of the TMR**

| Ingredient | C.P | TDN |
| --- | --- | --- |
| Jowar straw (50%) | 1.50 | 22.50 |
| Maize (11%) | 1.10 | 8.80 |
| Soyabean (13%) | 5.85 | 9.10 |
| DORB (17%) | 2.21 | 10.20 |
| Molasses (7%) | 0.21 | 3.85 |
| Mineral mixture, Salt (< 2 %) | 0.05 | 0.00 |
|  | **10.92** | **54.45** |

**Table S7. In vitro digestibility of total mixed rations (TMR) with varying levels of live fed microbials (LFM)**

| TMR with different levels of LFM | Jowar straw (%) | Concentrates (%) | LFM (%) |
| --- | --- | --- | --- |
| D_0_ | 50 | 50 | 0 |
| D_1_ | 50 | 50 | 1 |
| D_2_ | 50 | 50 | 2 |
| D_3_ | 50 | 50 | 3 |
| D_4_ | 50 | 50 | 4 |
| D_5_ | 50 | 50 | 5 |
| D_6_ | 50 | 50 | 6 |
| D_7_ | 50 | 50 | 7 |

**Figure S1.** In vivo methane yield in calves supplemented with LFM


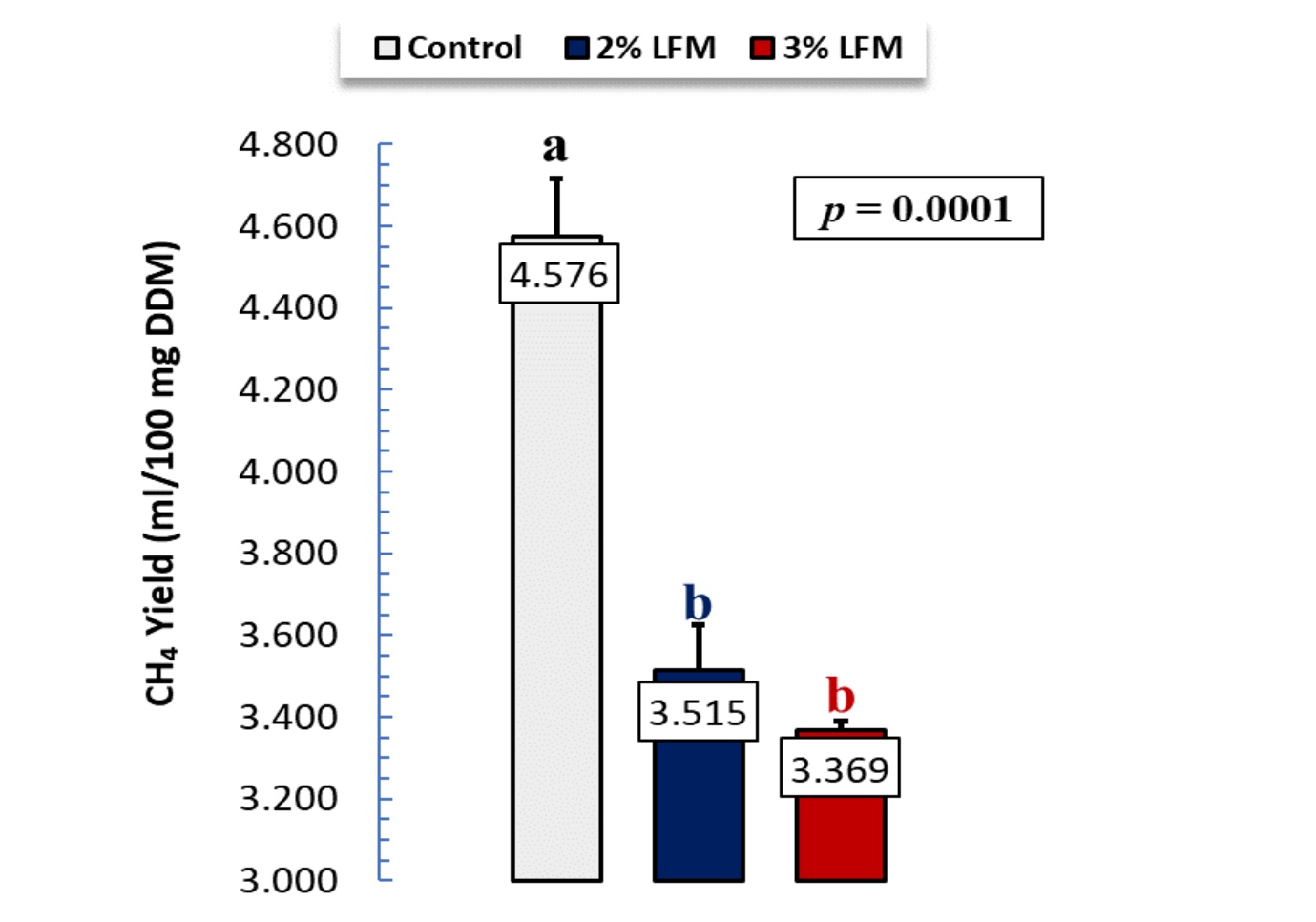


Superscripts (a, b) indicate statistically significant differences (p < 0.05).

**Figure S2.** Ingredient and nutrient composition of total mixed rations (TMR)


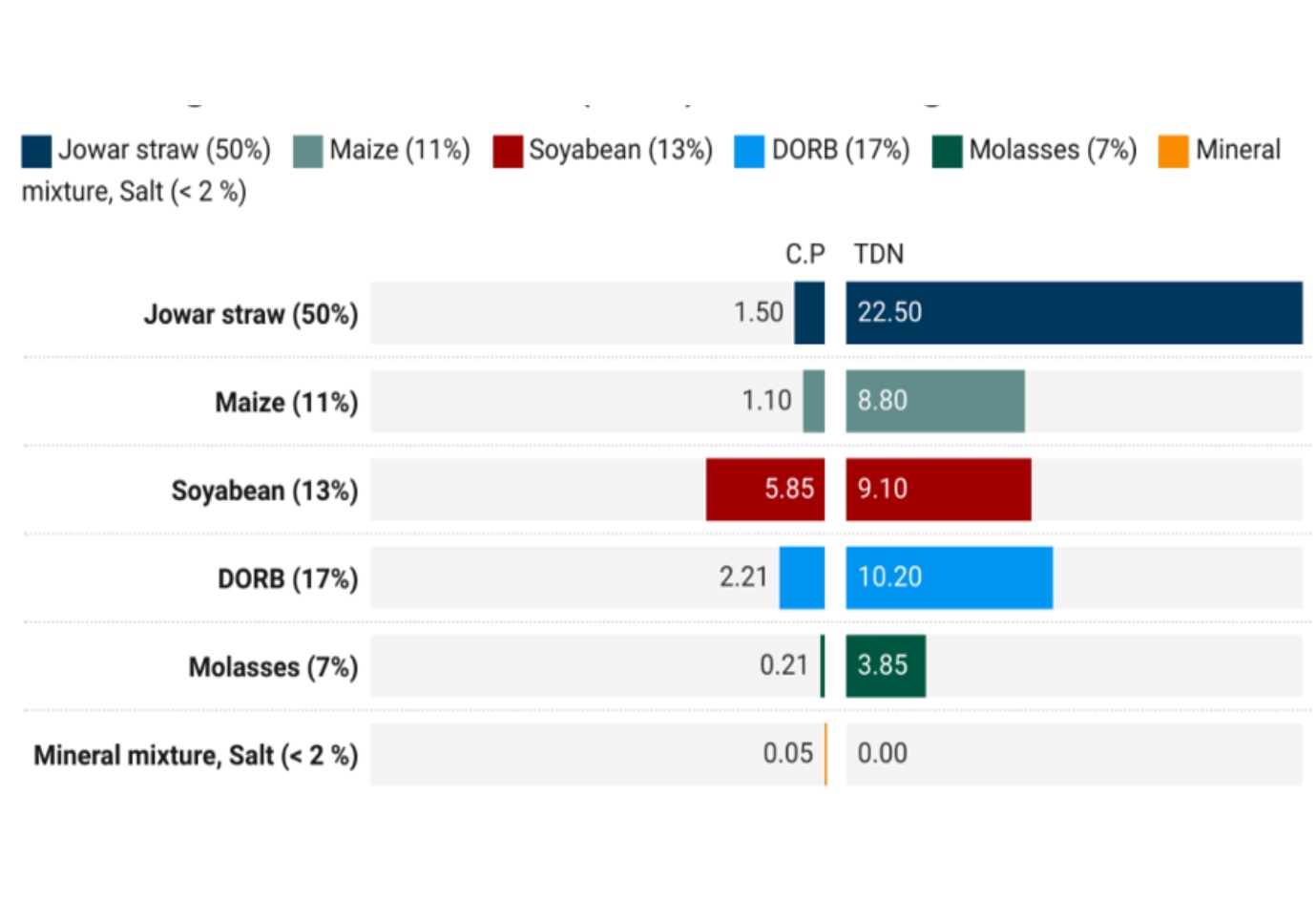


The Crude Protein (CP): Soybean contributes significantly to the CP value, making it the primary protein source, while Jowar Straw and DORB also contribute but to a lesser extent. The Total Digestible Nutrients (TDN): Jowar Straw and DORB are significant contributors to the energy value of the feed, while Maize and Molasses provide a moderate amount of TDN. Mineral Mixture has a negligible contribution to TDN.
